# Isolation of Cottonseed Extracts That Affect Human Cancer Cell Growth

**DOI:** 10.1038/s41598-018-28773-4

**Published:** 2018-07-11

**Authors:** Heping Cao, Kandan Sethumadhavan, John M. Bland

**Affiliations:** United States Department of Agriculture, Agricultural Research Service, Southern Regional Research Center, 1100 Robert E. Lee Boulevard, New Orleans, LA 70124 USA

## Abstract

Cottonseeds are classified as glanded or glandless seeds depending on the presence or absence of gossypol glands. Glanded cottonseed has anticancer property and glandless cottonseed was reported to cause cancer in one animal study. It is important to investigate the effect of bioactive components from cottonseeds. Our objectives were to isolate ethanol extracts from cottonseeds and investigate their effects on human cancer cells. A protocol was developed for isolating bioactive extracts from seed coat and kernel of glanded and glandless cottonseeds. HPLC-MS analyzed the four ethanol extracts but only quercetin was identified in the glandless seed coat extract. Residual gossypol was detected in the glanded and glandless seed kernel extracts and but only in the glanded seed coat extract. Ethanol extracts were used to treat human cancer cells derived from breast and pancreas followed by MTT assay for cell viability. Ethanol extracts from glanded and glandless cottonseed kernels and gossypol significantly decreased breast cancer cell mitochondrial activity. Ethanol extract from glanded cottonseed kernel and gossypol also significantly decreased pancreas cancer cell mitochondrial activity. These results suggest that ethanol extracts from cottonseeds, like gossypol, contain anticancer activities.

## Introduction

**Figure 1:**
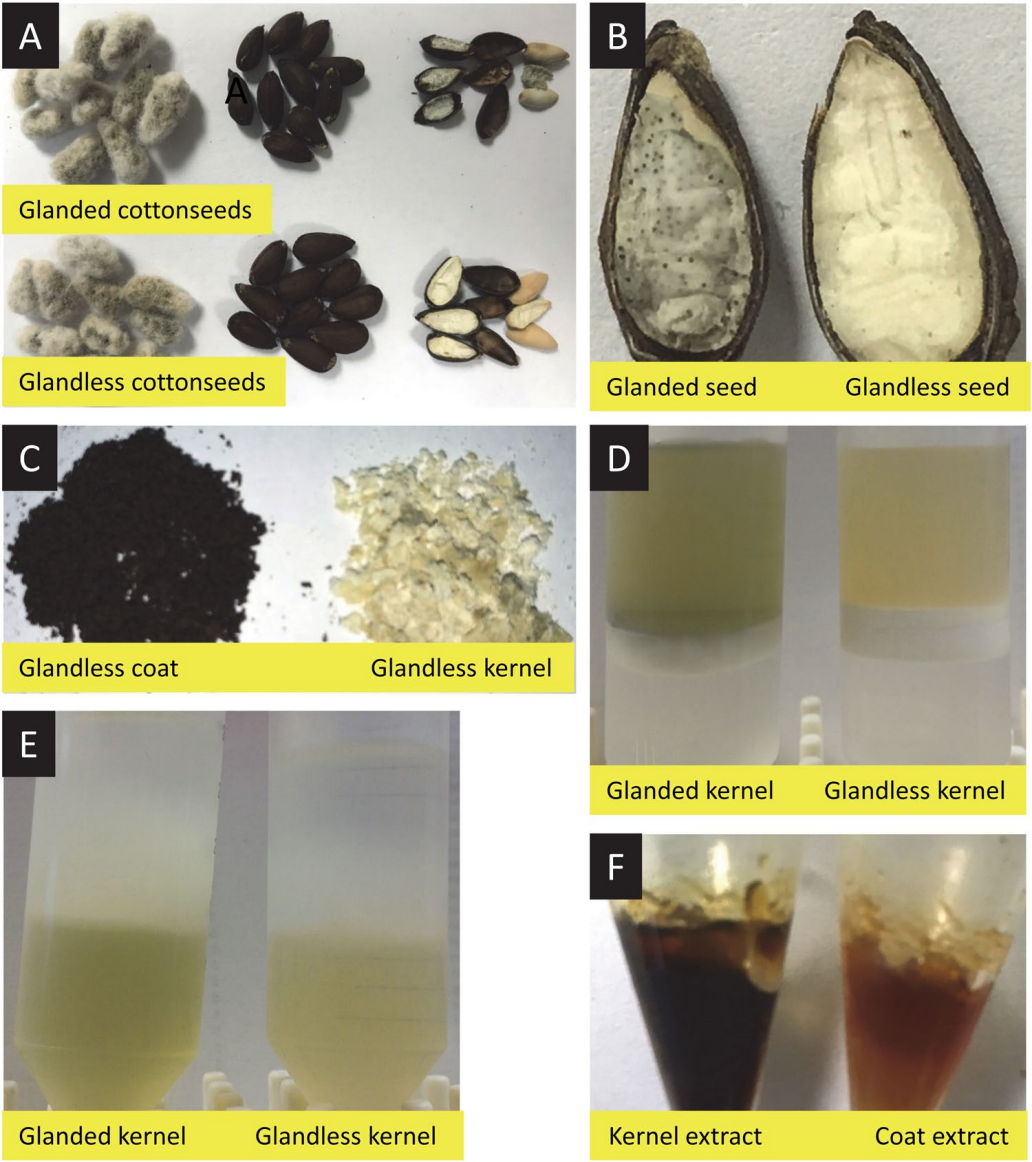
Glanded and glandless cottonseeds and isolation of ethanol extracts from the cottonseeds. (**A**) Glanded and glandless cottonseeds with short fibers, after sulfuric acid removal of the short fibers, and the seed coat and kernel. Both types of seeds were indistinguishable outside. (**B**) Section of glanded and glandless cottonseeds. Glanded seeds were smaller than glandless seeds and contained numerous dark green-colored glands in the kernel. (**C**) Glandless cottonseed coat and kernel. (**D**) Chloroform extraction. The glanded and glandless kernel homogenates were treated with chloroform followed by centrifugation to separate aqueous (upper) and organic (lower) layers. (**E**) Hexane extraction: The upper aqueous layer was mixed with hexane followed by centrifugation to separate neutral lipids (upper) and aqueous layers. (**F**) Ethanol extraction. Seed coat fraction after hexane extraction was suspended in acetic acid, blended, autoclaved and centrifuged. The supernatant was mixed with ethanol followed by centrifugation. The defatted kernel material was directly mixed with ethanol, vortexed and centrifuged. This supernatant was dried under rotoevaporation until all ethanol evaporated. The dried ethanol extracts were stored at −20 °C freezer.

Cotton (*Gossypium hirsutum* L.) is an industrially important crop because it provides fiber and cottonseeds. Cottonseeds account for only 20% of the crop value despite of being weighted much more than fiber in terms of mass. Cottonseeds are classified as either glanded or glandless seeds depending on the presence or absence of the dark pigment glands which contain polyphenolic gossypol (Fig. 1)^1–4^. Glanded cottonseeds contain approximately 10% linters, 40% hulls and 50% kernels^5^. Cottonseed kernels contain about 35% of oil and 40% of protein^6^. After oil extraction from the seeds, commercial cottonseed meal contains approximately 1% of gossypol^7^. The residual gossypol limits its use of cottonseed meal primarily to feed ruminants, which have a relative high tolerance for the toxic compound^8–12^. The ability of gossypol binding to protein also makes it more difficult to recover concentrated protein fractions from the meal free of gossypol^13^. Glandless cottonseeds lack pigment glands and have only trace levels of gossypol which may be useful for potential utilization of the protein as a food ingredient or as a feed for non-ruminant animals^14–17^. Therefore, development of glandless cotton has generated considerable interest within the cotton industry^18–22^.

Glanded cottonseeds contain a number of minor bioactive components. Gossypol is the best studied minor component from glanded cottonseed, Gossypol is a complex polyphenol with a highly colored yellow pigment found in the small intercellular pigment glands in cotton leaves, stems, roots, and seeds (Fig. 1A)^23^. Gossypol has been shown to have anti-nutritional property and potential biomedical applications. It has been known that long-term consumption of gossypol-containing cottonseed oil contributes to its toxicity resulting in male infertility^9^. Therefore, gossypol is regarded as unsafe for most animal and human consumption. Significant efforts have been directed to reduce gossypol content in cottonseeds by selecting glandless cotton varieties^13–17,24^ and genetic engineering of gossypol-free seeds of cotton plants^18–20^. However, recent studies have shown that gossypol and related compounds have anticancer activities, including breast cancer^25,26^, colon cancer^27^, pancreatic cancer^28,29^, and prostate cancer^30,31^. These new discoveries have generated intensive interest in biomedical field and enormous amounts of research have been directed at understanding the medical utilization of gossypol and related compounds.

Glandless cottonseeds contain trace amount of gossypol and are generally considered safe^13–15^. Glandless cottonseeds and their modified products have been approved for human consumption as a nut substitute and snack item by the Food and Drug Administration (https://www.gpo.gov/fdsys/pkg/CFR-2012-title21-vol3/pdf/CFR-2012). These seeds may be available for consumption by human and non-ruminant animals in the future^17,18^. However, glandless cottonseeds contain other growth inhibitors^32^ such as cyclopropenoid fatty acids, which caused liver cancer in rainbow trout in one study^24^.

Considering the conflicting results mentioned above that glanded cottonseeds, rich in gossypol, may have anticancer property and glandless cottonseeds essentially free of gossypol may cause cancer in animals, it is important to investigate the effect of bioactive components from cottonseeds in cancer cells. Therefore, the objectives of this study were to isolate bioactive extracts from glanded and glandless cottonseeds and investigate their effects on cultured human cancer cells using gossypol and lipopolysaccharides (LPS) as controls.

## Results

### Isolation of Ethanol Extracts from Cottonseeds

**Figure 2:**
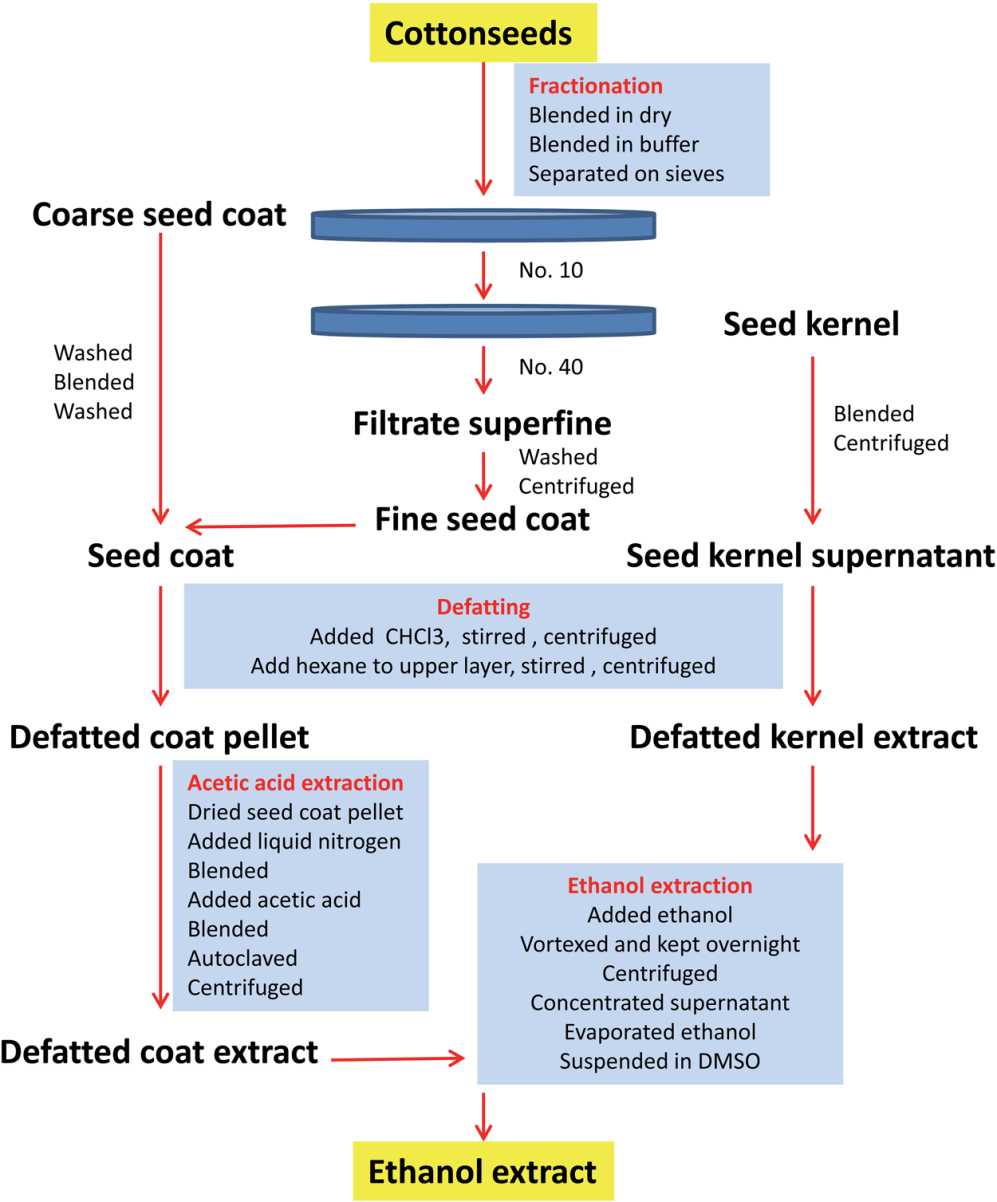
The protocol for isolating ethanol extracts from cottonseeds. This method was consistent of three steps for seed kernel extraction (fractionation, defatting, and ethanol extraction) and four steps for seed coat extraction (fractionation, defatting, acetic acid extraction, and ethanol extraction). Briefly, cottonseeds were ground in dry and in a buffer. The homogenate was separated successively through No. 10 sieve to retain coarse seed coat and No. 40 sieve to retain fine seed kernel and colored superfine filtrate. The coarse seed coat was washed with water and blended several times until clear seed coat pellet was obtained. The colored superfine filtrate was mixed with water and allowed to stand and centrifuged. The red pellet of fine seed coat was pooled together with the coarse seed coat and suspend in the buffer. The seed kernel was suspended in the same buffer followed by grinding and centrifugation. The seed coat suspension and kernel supernatant were defatted with chloroform and hexane followed by centrifugation. Defatted seed coat pellet was air-died after defatting and grinded into fine powder under liquid nitrogen. The fine powder was suspended in acetic acid, ground again, autoclaved, and centrifuged. The supernatant was filtrated through glass wool, mixed with ethanol, stirred well, refrigerated overnight and centrifuged. The supernatant was concentrated in a rotovap and residue ethanol was removed by rotoevaporation. The preparation of ethanol extract from the defatted kernel extract was similar to those of defatted coat extract without acetic acid treatment. The dried ethanol extract pellet was reconstituted in DMSO.

The purpose of selecting ethanol extract in this study was because previous research showed that plant polyphenols with nutritional values were mostly water-soluble and could be extracted by ethanol from plant materials such as cinnamon bark and green tea leaves and that some toxic compounds such as cinnamaldehyde (essential oil) could be extracted from cinnamon bark by organic solvent^33–36^. The protocol for isolating ethanol extracts from cottonseeds is summarized in Figs 1 and 2. This method was involved in three steps for ethanol extraction from seed kernel (fractionation, defatting, and ethanol extraction) and four steps for ethanol extraction from seed coat (fractionation, defatting, acetic acid extraction, and ethanol extraction). Briefly, the seed coat and kernel of glanded and glandless cottonseeds were fractionated by grinding and homogenization (Fig. 1A–C). The seed kernel fractions were defatted with equal volume of chloroform and hexane followed by centrifugation. The aqueous layer from glanded kernel exhibited much darker/greener color than that from glandless kernel (Fig. 1D,E). The seed coat fraction was suspended in acetic acid, autoclaved and centrifuged before ethanol extraction. The ethanol extracts were dried under rotoevaporation until all acetic acid and ethanol evaporated. The seed kernel extract was much darker in color than the seed coat extract (Fig. 1F). This procedure yielded 0.39 g of ethanol extract from seed coat and 3.66 g of ethanol extract from seed kernel per 100 g of glanded cottonseeds, and 0.98 g of ethanol extract from seed coat and 1.12 g of ethanol extract from seed kernel per 100 g of glandless cottonseeds.

### HPLC-MS Analysis of Ethanol Extracts from Cottonseeds

**Figure 3:**
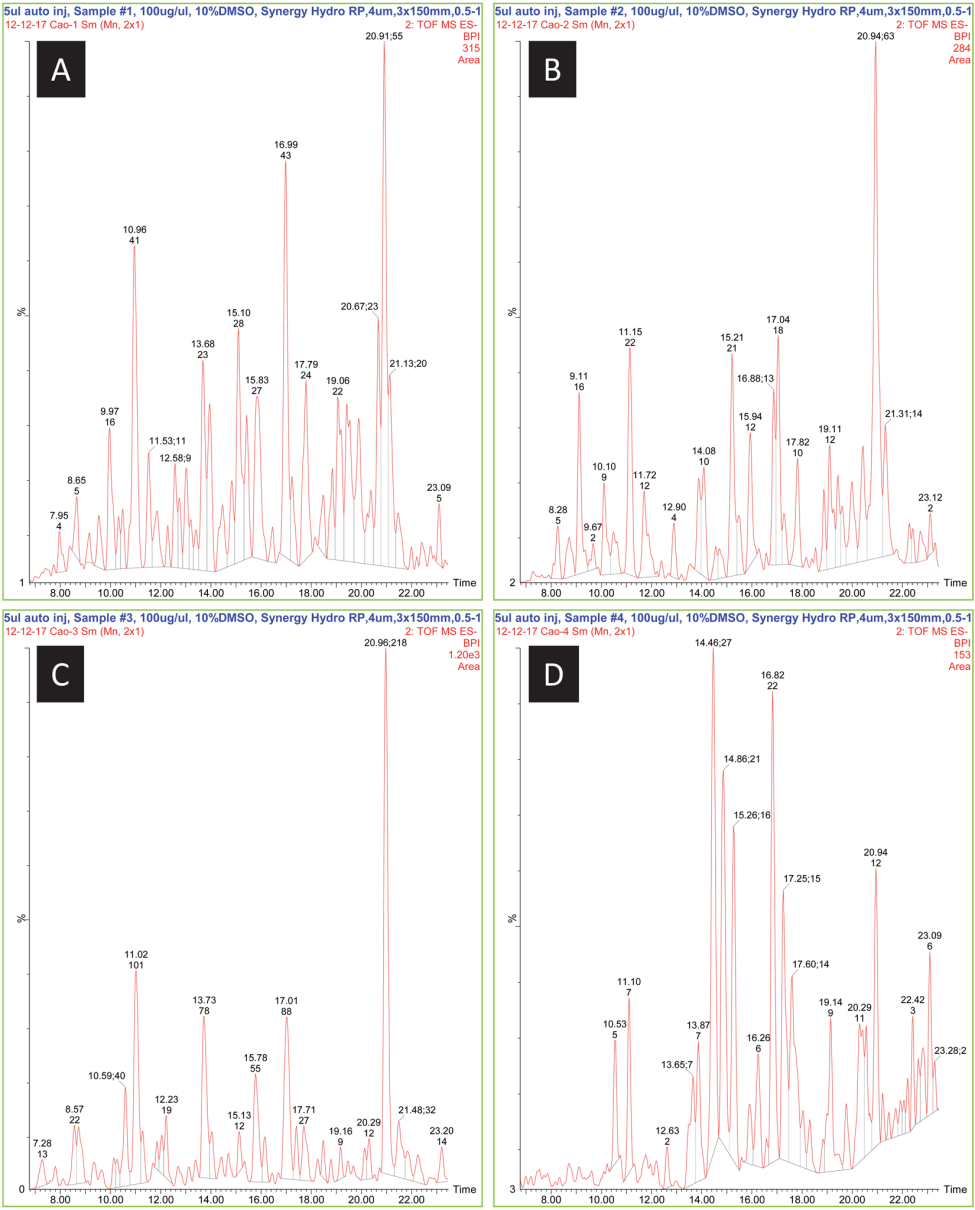
HPLC-MS analysis of ethanol extracts from cottonseeds. HPLC-UV-MS analyzed the four ethanol extracts from glanded and glandless cottonseeds using 5 μl injection of a 10% DMSO solution of the extract. (**A**) glanded cottonseed coat extract, (**B**) glanded cottonseed kernel extract, (**C**) glandless cottonseed coat extract, (**D**) glandless cottonseed kernel extract.

**Figure 4:**
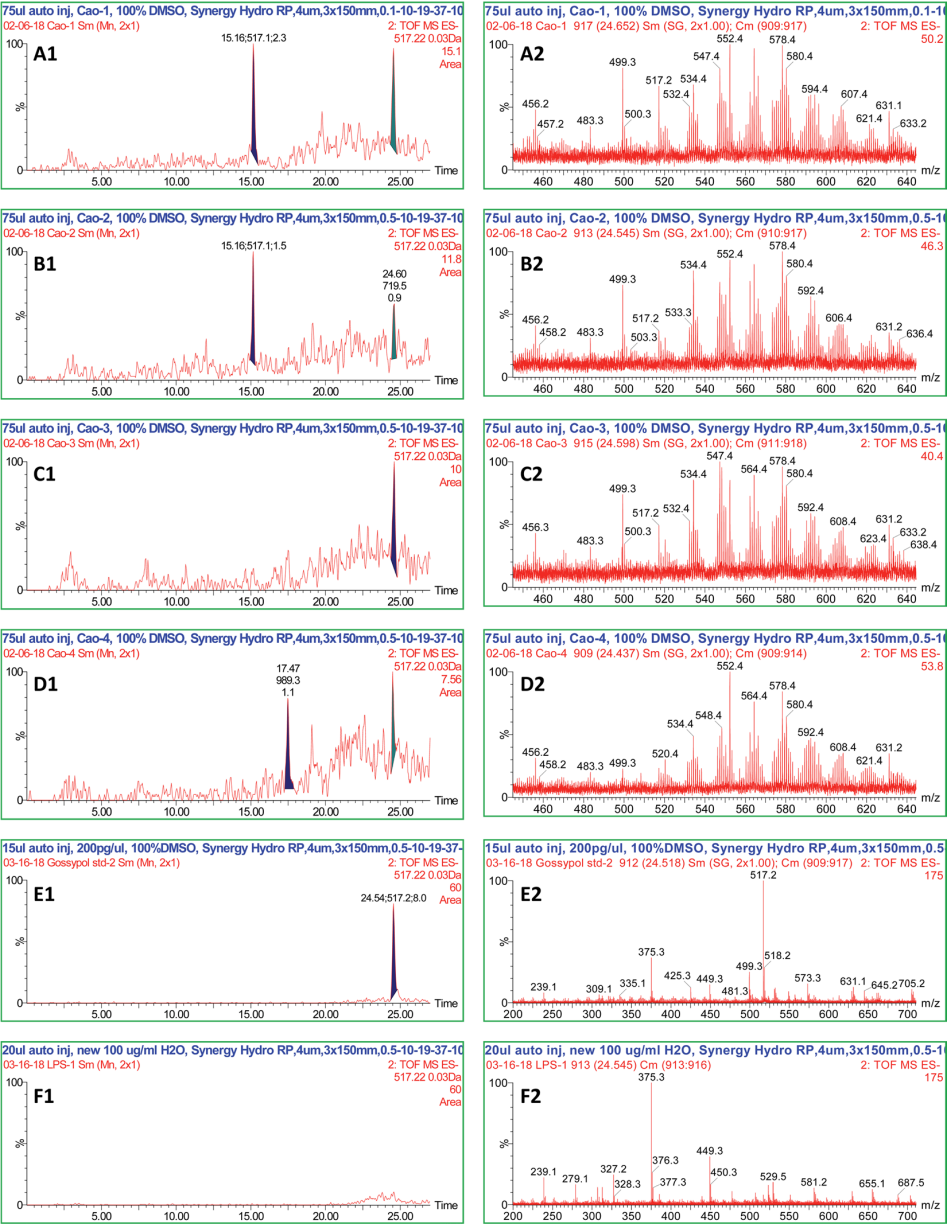
Detection of gossypol in ethanol extracts from cottonseeds. HPLC-UV-MS analyzed the four ethanol extracts from glanded and glandless cottonseeds using 75 μl injection of a 100% DMSO solution of the extract. (**A**) glanded cottonseed coat extract, (**B**) glanded cottonseed kernel extract, (**C**) glandless cottonseed coat extract, (**D**) glandless cottonseed kernel extract, (**E**) gossypol stand, (**F**) LPS. Left panels: chromatogram of gossypol ion mass at 517.22 (M-H)^−^ with retention time of 24.5 min. Right panels: full mass spectrum.

HPLC-UV-MS analyzed the four ethanol extracts from glanded and glandless cottonseeds using 5 μl injection of a 10% DMSO solution of each extract (Fig. 3). Negative ionization (ESI-neg) was determined to be preferential for flavonols, however confirmation in positive ionization (ESI-pos) and UV were used to qualify flavonol peaks. A large number of peaks were observed in ESI-neg, with 27 peaks having mass values equivalent to possible flavonols or derivatives. Because only 11 flavonol standards were available for comparison, only quercetin could be positively identified by retention time, ESI-neg, and ESI-pos, but not by UV. The other 10 flavonol standards were not detected, based on retention times. The other 16 possible matches to apiosyl, rhamnosyl, and glucosyl-derivative masses could not be verified. The quercetin peak was only identified in the glandless seed coat. Gossypol was quantified in extracts from cottonseed coat and kernels and LPS (Fig. 4). The left panels showed the chromatogram of gossypol ion mass at 517.22 (M-H)^−^ with retention time of 24.5 min, whereas the right panels showed the full mass spectrum of the samples. Seed coats from glanded (Fig. 4A) and glandless seeds (Fig. 4C) contained 8.2 and 3.7 pg/µl of gossypol, respectively. Seed kernels from glanded (Fig. 4B) and glandless seeds (Fig. 4D) contained 0.3 and 0 pg/µl, respectively. Compared to standard gossypol (Fig. 4E), no gossypol was found in the LPS (Fig. 4F).

### Effect of Cottonseed Extracts, Gossypol and LPS on Breast Cancer Cell Growth

**Figure 5:**
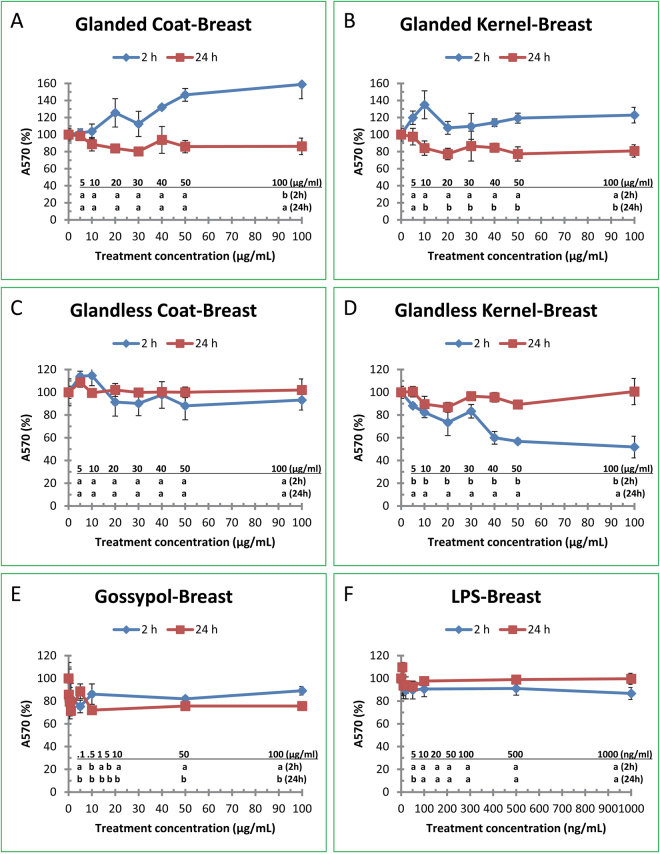
Effect of cottonseed extracts, gossypol, and LPS on human breast cancer cell growth. Human breast cancer cells (MCF7) were treated with various concentrations of cottonseed extracts and chemicals for 2 and 24 h. The cell media were added with MTT assay reagent, and incubated for 2 h before adding MTT solubilization solution, shaken at room temperature overnight. The color density in the wells was recorded at A570. The data represent the mean and standard deviation of three independent samples. Values with different lower case letters compared to the DMSO control (treatment concentration = 0) in the lower part of the Figure are significantly different at *p* < 0.05 among the various treatment concentrations in 2 or 24-h treatment time. (**A**) glanded cottonseed coat extract, (**B**) glanded cottonseed kernel extract, (**C**) glandless cottonseed coat extract, (**D**) glandless cottonseed kernel extract, (**E**) gossypol, (**F**) LPS.

Cell cytotoxicity of human breast cancer cells (MCF7) was determined with MTT method after breast cancer cells were treated for 2 h and 24 h with 5–100 µg/ml of ethanol extracts from the coat and kernel of glanded and glandless cottonseeds (Fig. 5). Figure 5A shows that the cell viability was significantly increased up to 60% by extract from glanded cottonseed coat with 100 µg/ml treatment for 2 h but declined, statistically insignificantly, by 15% after treatment for 24 h. However, extract from glanded cottonseed kernel significantly reduced the cell viability by 25% after 24 h treatment with 10–100 µg/ml concentrations (Fig. 5B). MTT assays showed that extracts from glandless cottonseed coat did not have significant effect on breast cancer cell viability after treatment for 2 h or 24 h with 5–100 µg/ml of the extract concentrations (Fig. 5C) but glandless kernel extract significantly reduced cell viability by up to 50% after 5–100 µg/ml treatment for 2 h although it did not affect breast cell viability after 24 h treatment (Fig. 5D). Similar experiments were conducted using LPS and gossypol. MTT assays showed that gossypol significantly decreased breast cancer cell growth by approximately 30% after 24 h treatment (Fig. 5E), but LPS did not have much effect on breast cell growth (Fig. 5F).

### Effect of Cottonseed Extracts, Gossypol and LPS on Pancreatic Cancer Cell Growth

**Figure 6:**
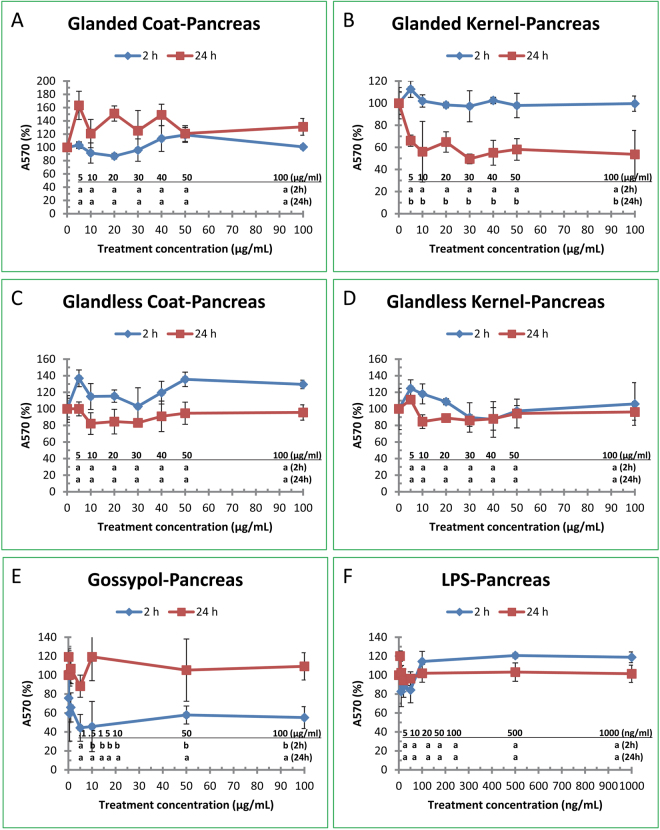
Effect of cottonseed extracts, gossypol, and LPS on human pancreatic cancer cell growth. Human pancreatic cancer cells (MIA PaCA-2) were treated with various concentrations of cottonseed extracts and chemicals for 2 and 24 h. The cell media were added with MTT assay reagent, and incubated for 2 h before adding MTT solubilization solution, shaken at room temperature overnight. The color density in the wells was recorded at A570. The data represent the mean and standard deviation of three independent samples. Values with different lower case letters compared to the DMSO control (treatment concentration = 0) in the lower part of the Figure are significantly different at *p* < 0.05 among the various treatment concentrations in 2 or 24-h treatment time. (**A**) glanded cottonseed coat extract, (**B**) glanded cottonseed kernel extract, (**C**) glandless cottonseed coat extract, (**D**) glandless cottonseed kernel extract, (**E**) gossypol, (**F**) LPS.

Pancreatic cancer cell (MIA PaCA-2) was also used to test the effects of cottonseed extracts on cancer cell growth. The cells were treated with ethanol extracts from coat and kernel of glanded and glandless cottonseeds (5–100 µg/ml) for 2 h and 24 h (Fig. 6). Figure 6A shows that the cell viability was not significantly affected by extract from glanded cottonseed coat. Similarly, extract from glanded cottonseed kernel did not affect cancer cell growth by 2 h treatment. However, extract from glanded kernel significantly decreased pancreatic cancer cell viability by approximately 50% after 24 h treatment (Fig. 6B). Similar experiments were conducted on human pancreatic cancer cell viability using extracts from glandless cottonseed coat and kernel. MTT assays showed that extracts from glandless cottonseed coat or kernel did not have significant effects on pancreatic cancer cell viability (Fig. 6C,D). The effect of gossypol treatment for 2 h significantly decreased cell viability by up to 50% (Fig. 6E). However, LPS on the pancreatic cancer cell growth was minimal (Fig. 6F).

## Discussion

Cottonseed accounts for approximately 20% of the crop value. However, cottonseed usage is limited to feeding ruminant animals and not for human or other animal consumption due to toxic gossypol in glanded cottonseed, the commonly cultivated cotton^8–12^. Glandless cottonseed does not have pigment glands and accumulates only trace amounts of gossypol^17^; which has potential being used as a food ingredient or as a feed for non-ruminant animals^14–16^. To explore additional value of cottonseeds, we isolated bioactive extracts from both glanded and glandless cottonseeds and tested their bioactivity towards human cancer cells.

The novelty of this paper is the development of a reliable protocol for isolating bioactive extracts from the seed coat and kernel of glanded and glandless cottonseeds. The method was consistent of three steps for ethanol extraction from seed kernel (fractionation, defatting, and ethanol extraction) and four steps for ethanol extraction from seed coat (fractionation, defatting, acetic acid extraction, and ethanol extraction). The overall yield of ethanol extracts was approximately 4% and 2% from 100 g of glanded and glandless cottonseeds, respectively. HPLC-UV-MS analyzed the four ethanol extracts from glanded and glandless cottonseeds. We observed 27 peaks having mass values equivalent to possible flavonols or derivatives and 16 peaks possible matched to apiosyl, rhamnosyl, and glucosyl-derivative masses but only quercetin peak was identified in the glandless seed coat extract. Quercetin was reported to have antidepressant effect^37^. A similar protocol was used to isolate ethanol extracts from cinnamon bark which was shown to be polyphenol mixtures^34,36^. Cinnamon polyphenolic extract was shown to have a wide range of bioactivity in mouse adipocytes and macrophages^36,38–40^. Although the exact composition of ethanol extracts from cottonseeds was not determined in this report, it is speculative that they are probably also polyphenol components in cottonseed extracts. Additional experiments are required to identify the bioactive components and correlate them to specific bioactivity towards cancer cells. In addition, different extraction protocol may yield different bioactive compounds from cottonseeds.

The important finding of this study is that ethanol extracts from cottonseeds can regulate human cancer cell growth. Specifically, ethanol extracts from glanded and glandless cottonseed kernels and gossypol significantly decreased breast cancer cell mitochondrial activity. Ethanol extract from glanded cottonseed kernel and gossypol also significantly decreased pancreas cancer cell mitochondrial activity. Ethanol extract from glanded cottonseed coat increased mitochondrial activity in breast cancer cells by short-term treatment but its effect returned to normal after longer-term treatment. Ethanol extract from glanded cottonseed kernel significantly decreased mitochondrial activity in breast cancer cells after 24-h treatment. In contrast, ethanol extract from glandless kernel but not from seed coat significantly decreased mitochondrial activity of the breast cancer cells after 2-h treatment. Gossypol but not LPS also significantly decreased mitochondrial activity of these cells. The effect of gossypol on decreasing breast cancer cell (MCF-7) was in agreement with previous observations using gossypol, gossypol derivative, and gossypol-enriched cottonseed oil^41–46^. The inhibitory effect of extract from glanded cottonseed kernel could be explained by the residual gossypol in the extract. However, it was difficult to explain why glandless cottonseed kernel extract also rapidly decreased breast cancer cell viability. Furthermore, glanded cottonseed coat extract increased mitochondrial activity in the breast cancer cell. It is speculative that other bioactive compound(s) was presented in the glandless kernel extract and glanded coat extract which may have properties of anti- and pro-breast cancer cell growth. In pancreatic cancer cells, 24-h treatment of ethanol extract from glanded seed kernel significantly decreased cellular mitochondrial activity. Gossypol also significantly decreased cellular activity after short-term treatment which is in agreement with published reports showing inhibitory effect of gossypol and its derivatives on pancreatic cancer cell viability^28,47–49^.

The above results suggest that anti-cancer components are present in the cottonseed kernel extracts and pro-cancer compound might be present in the glanded cottonseed coat extract. It is also possible that pro- and anti-cancer activity of cottonseed extracts was due to effects of multiple compounds in the cottonseed extracts. Besides the well-studied gossypol, other compounds from glanded cottonseed exhibit significant bioactivities, such as gallic acid and 3,4-dihydroxybenzoic acid that have positive effects on health^50^. Gossypol has additional bioactivities such as antiobesity activities^25,51^, antiinflammatory activities^52,53^, and antifungal activities^54,55^. Multiple bioactive components have been identified in glandless cottonseed. Flavonol glycosides (flavonoids) are the best studied compounds from glandless cottonseeds. It was reported that aqueous extract from glandless cottonseed meal had antidepressant effect and subsequently the authors identified the major bioactive compound being quercetin 3-*O*-ß-D-apiofuranosyl-(1 → 2)-[α-L-rhamnopyranosyl-(1 → 6)]-ß-D-glucopyranoside (CTN-986)^37^. This compound was shown to have antidepressant effects in pharmacological tests^56,57^ and have potential applications in treating anxiety, depression and Alzeheimer’s disease^58^. Five flavonoids have been identified from glandless cottonseeds^37,59^. Seven flavonol glycosides have also been identified from whole cottonseeds^50^. Independent studies have confirmed that flavonoids could be used as a therapy for depression associated with diabetes^60^ and under other conditions^61^.

It is unknown about the molecular mechanism(s) of anti-cancer effects of ethanol extracts from cottonseed in the current investigation. It has been shown that gossypol inhibits breast cancer cells via DNA synthesis^42^, suppressing Bcl-2 and Bcl-xL expression^44^. Gossypol also inhibits Bcl-2 and Mcl-1 gene expression in pancreatic cancer cells^47^. However, we can only speculate that ethanol extracts on inhibiting cancer cell viability was probably due in part to the residual gossypol effect. Additional research will be required if other compounds in the ethanol extracts independently inhibit cancer cell viability, and if so, what kind of mechanism(s) is involved in the process.

In conclusion, we reported the isolation of ethanol extracts from the coat and kernel of glanded and glandless cottonseeds and tested their bioactivity in human cancer cells. The method was consistent of three steps for seed kernel extraction (fractionation, defatting, and ethanol extraction) and four steps for seed coat extraction (fractionation, defatting, acetic acid extraction, and ethanol extraction) with 2–4% yield. HPLC-UV-MS analyzed the four ethanol extracts from glanded and glandless cottonseed with 27 peaks having mass values equivalent to possible flavonols or derivatives and 16 peaks possible matched to apiosyl, rhamnosyl, and glucosyl-derivative masses but only quercetin peak was identified in the glandless seed coat extract. Residual gossypol was detected in the kernel extracts of glanded and glandless seed and but only in the coat extract of glanded seed. Bioactivity study showed that ethanol extracts from cottonseeds decreased the mitochondrial activity of cancer cell lines derived from breast and pancreas. Further studies are required to identify the specific component(s) that has anti-cancer properties. We suggest that cottonseed value could be increased by exploring bioactivity of minor components in the seeds which have potential health and nutritional benefits for cancer-related diseases.

## Materials and Methods

### Cottonseeds

Glanded cottonseeds from variety “DP1321” were donated by Richard K. Byler (USDA-ARS, Stoneville, MS). Glandless cottonseeds from variety “NuMex 15 GLS” were donated by Thomas C. Wedegaertner (Cotton, Inc., Cary, NC)^21^. Cotton bolls were fed into a gin machine to separate the long fibers from the seeds (Fig. 1A). The short fibers on the seeds (also called linters) were further removed from the seeds with sulfuric acid using the following procedure. Cottonseeds were transferred into a beaker and covered with concentrated sulfuric acid. The contents in the beaker were mixed with a glass rod and kept for 2 min at room temperature followed by filtration through metallic wire gauze. This procedure was repeated until there was no fiber on the seeds (Fig. 1A). The seeds were washed with distilled water extensively followed by neutralizing any residual acid with sodium bicarbonate solution. After thorough washing in distilled water, the seeds were dried on a paper towel on the bench top and stored at room temperature until being used. Both types of seeds were indistinguishable outside, but glanded seeds were smaller than glandless seeds and contained numerous dark-colored glands in the kernel (Fig. 1B).

### Cancer Cell Lines

Two human cancer cell clines were used in the experiments. They were human breast cancer cell line (MCF7, ATCC HTB-22) and pancreatic cancer cell line (MIA PaCA-2, ATCC CRL-1420). These cancer cell lines were purchased from American Type Culture Collection (Manassas, VA) and kept under liquid nitrogen vapor in a Cryogenic Storage Vessel (Thermo Fisher Scientific, Waltham, MA).

### Chemicals, Reagents and Equipment

Cell cytotoxicity reagent (MTT based-*In Vitro* Toxicology Assay Kit), gossypol, LPS and other chemicals (chloroform, DMSO, ethanol, hexane, sodium bicarbonate, sulfuric acid) were from Sigma (St. Louis, MO). Tissue culture reagents (DMEM, MEM, fetal bovine serum, penicillin, streptomycin, L-glutamine) were from Gibco BRL (Thermo Fisher). Tissue culture incubator was water jacket CO_2_ incubator, Forma Series II, Model 3100 Series (Thermo Fisher). Tissue culture workstation was Logic + A2 hood (Labconco, Kansas City, MO). Tissue culture plastic ware (flasks, plates, cell scraper) was from CytoOne (USA Scientific, Ocala, FL). Cell counting reagent (trypsin blue dye), slides (dual chamber), counter (TC20 Automatic Cell Counter) and microscope (Zoe Florescent Cell Imager) were from Bio-Rad (Hercules, CA). Molecular sieves were from USA Standard Testing Sieves (Milwaukee, WI). Microplate spectrophotometer (Epoch) was from BioTek Instruments (Winooski, VT).

### Fractionation of Cottonseed Coat and Kernel

Cottonseeds from glanded and glandless cotton were ground into pieces by a Waring commercial blender 7010 (Torrington, CT) (Fig. 2). The pieces of seeds were ground again in a homogenization buffer (1.5 mL/g seeds) containing 50 mM Tris-HCl and 150 mM NaCl, pH 7.4. The homogenate was separated successively through No. 10 sieve to retain coarse material (mostly seed coat) and No. 40 sieve to retain fine material (mostly kernel) and colored superfine filtrate. The coarse material from No. 10 filtration (mostly seed coat) was washed with water and blended several times until clear seed coat pellet was obtained. The colored superfine filtrate from No. 40 filtration was mixed with water and allowed to stand and centrifuged at 7,150 *g* for 20 min. This red pellet of fine seed coat was pooled together with the seed coat from the coarse material and air-dried for defatting, acetic acid treatment and ethanol extraction. The fine material from No. 40 sieve (mostly kernel) was washed with the homogenization buffer and suspended in the same homogenization buffer followed by grinding. The homogenate was centrifuged at 7,150 *g* for 20 min. The supernatant was used for seed kernel defatting and ethanol extraction.

### Defatting of Cottonseed Coat and Kernel

The fractions from cottonseed coat and kernel were defatted by successive extraction with chloroform and hexane according to a procedure essentially as described previously^50^. Briefly, the seed coat suspension and kernel supernatant were defatted with equal volume of chloroform by stirring the mixture for 30 min followed by centrifugation at 3,400 *g* for 20 min. The upper aqueous layer was transferred into a fresh tube and mixed with an equal volume of hexane by stirring for 30 min followed by centrifugation as above to remove neutral lipids (Fig. 2).

### Ethanol Extraction of Cottonseed Coat and Kernel

Cottonseed coat extract was prepared basically following a procedure as described previously for cinnamon extract^34,36^. Cottonseed coat fraction was air-died after defatting and ground into fine powder under liquid nitrogen (Fig. 2). The fine powder was suspended in 250 ml of 0.2 N acetic acid, ground again and autoclaved for 15 min at 121 °C. The mixture was centrifuged at 7,150 *g* for 20 min. The supernatant was filtrated through glass wool, mixed with 1000 ml ethanol, stirred well, refrigerated overnight and centrifuged at 7,150 *g* for 30 min. Acetic acid was used to loosen the cell wall of cottonseed coat for increasing ethanol extraction efficiency^34^. The supernatant was concentrated in a rotovap and residual acetic acid and ethanol was removed by rotoevaporation using an Integrated SpeedVac System or freeze-drying at 30 °C and 90 bar until all ethanol evaporated. The preparation of ethanol extract from the defatted kernel extract (200 ml) was similar to those of defatted coat extract without acetic acid treatment (Fig. 2). The dried ethanol extract pellet was weighed and reconstituted at 100 mg/ml in 100% dimethylsulfoxide (DMSO).

### HPLC-MS Analysis

The four ethanol extracts from glanded and glandless cottonseed were analyzed by HPLC-UV-MS on a Waters Alliance (2695), PDA detector (996), and a Waters LCT Premier-XE mass spectrometer (ToF) with an electrospray ionization (ESI) source. UV was detected at 210–600 nm. MS was detected at 200–1000 m/z. ESI conditions were: positive mode: 3500 V capillary voltage, 70 V cone voltage, 350 °C desolvation temperature, 105 °C source temperature, 450 l/min desolvation gas, 50 l/min cone gas; negative mode: 3000 V capillary voltage. A Synergy Hydro RP 4 µm 3.0 × 150 mm column (Phenomenex, Torrance, CA) was used with a gradient of acetonitrile/0.1% formic acid from 0.5% to 10% acetonitrile over 2 min, followed by a gradient to 19% over 9 min, to 37% over 3 min, and to 80% over 3 min. Equilibration at initial conditions was for 10 min between runs. Flow rate was 0.3 ml/min and column temperature was 30 °C. A 75 μl injection of a 100% DMSO solution of the extract was analyzed. Gossypol standard in DMSO was prepared at 2 pg/µl and LPS standard in water was prepared at 100 ng/µl. A standard curve was determined for gossypol for quantification based on peak area of its m/z 517.22 ± 0.03 in the negative ESI chromatogram.

### Cell Culture and Chemical Treatment

The basic cell culture protocol was following previous procedures^39,40,62^. Human breast cancer cell line (MCF7) and pancreatic cancer cell line (MIA PaCA-2) were maintained in 75 cm^2^ polystyrene tissue culture flasks as described^63^ at 37 °C in a water jacket CO_2_ incubator with 5% CO_2_ in MEM and DMEM, respectively. The media were supplemented with 10% (v/v) fetal bovine serum, 100 units/ml penicillin, 100 µg/ml streptomycin, and 2 mM L-glutamine. Cancer cells were dissociated from the T-75 flask with 0.25% (w/v) trypsin-0.53 mM EDTA solution, stained with equal volume of 0.4% trypsin blue dye before counting the number of live cells with a TC20 Automatic Cell Counter. Cancer cells (0.5 ml) from trypsin-dissociated flasks were subcultured at approximately 1 × 10^5^ cells/ml density in 24-well tissue culture plates. The cancer cells were routinely observed under a Zoe Florescent Cell Imager before and under treatment. Cancer cells were treated with 0, 5, 10, 20, 30, 40, 50, and 100 µg/ml of ethanol extracts, 0, 0.1, 0.5, 1, 5, 10, 50, and 100 µg/ml of gossypol, and 0, 5, 10, 20, 50, 100, 500, and 1000 ng/ml of LPS for 2 and 24 h (“0” treatment corresponding to 1% DMSO in the culture medium, the same concentration used to suspend the chemicals).

### Cell Viability Assay

Cell cytotoxicity was determined with the MTT based-*In Vitro* Toxicology Assay Kit. Cancer cells were treated with cottonseed extracts and incubated at 37 °C, 5% CO_2_ for 2 and 24 h. The cell media were added with 50 µl of MTT assay reagent (thiazolyl blue tetrazolium bromide) and incubated at 37 °C, 5% CO_2_ for 2 h before adding 500 µl MTT solubilization solution to each well, shaken at room temperature overnight. The color density in the wells was recorded by Epoch microplate spectrophotometer at A570 after diluting 8 times.

### Statistics

The data in the Figs represent the mean and standard deviation of three independent samples. They were analyzed by statistical analysis using ANOVA with SigmaStat 3.1 software (Systat Software). Multiple comparisons among the treatments with different concentrations of cottonseed extracts, gossypol and LPS were performed with Student-Newman-Keuls Method^40^.
